# Clinically Recognized Depression and Mental Health Treatment in a Single Center Cohort of Patients with Systemic Sclerosis

**DOI:** 10.1155/2023/6141790

**Published:** 2023-12-19

**Authors:** Marissa B. Savoie, Alexandra Poeschla, Na Lu, Yuqing Zhang, Marcy B. Bolster, Sara Schoenfeld, Flavia V. Castelino

**Affiliations:** ^1^Department of Medicine, Massachusetts General Hospital, USA; ^2^Department of Medicine, Division of Rheumatology, Allergy, and Immunology, Massachusetts General Hospital, USA; ^3^Arthritis Research Canada, Canada

## Abstract

**Introduction:**

In this study, we investigated the prevalence of depression, depression treatment, and symptom burden in patients with systemic sclerosis (SSc) and examined their associations with the center for epidemiologic studies depression scale revised (CESD-R) scores.

**Methods:**

The Prospective Registry in Scleroderma at Massachusetts General Hospital (PRISM) is a longitudinal registry of patients with SSc. Among participants with CESD-R score ≥ 16, indicating possible depression, a chart review was performed for mental health diagnoses and treatments. We examined the relation of demographic and clinical factors to the presence of mental health diagnoses or treatment using logistic regression. We evaluated the association of SSc symptoms and the COVID-19 pandemic with a CESD-R score using quantile regression.

**Results:**

Of 214 patients enrolled in PRISM, 129 participants (38% diffuse and 59% limited) completed at least one CESD-R questionnaire. In the first survey, 29% had possible depression (CESD − R ≥ 16) and 16% had probable depression (CESD − R ≥ 23). Of 20 participants with probable depression, 90% received treatment for a mood disorder. In a multivariable logistic regression model among participants with CESD − R ≥ 16, none of the evaluated variables (CESD-R score, age, gender, employment status, race, and ethnicity) was associated with mental health diagnosis or treatment. Higher baseline dyspnea index, modified Rodnan skin score, and the University of California Los Angeles Scleroderma Clinical Trials Consortium Gastrointestinal total score and subscores were associated with higher CESD-R score.

**Conclusion:**

In this single-center cross-sectional study, 16% of participants had significant depressive symptoms. Dyspnea, extent of skin involvement, and gastrointestinal symptoms were associated with depression symptoms.

## 1. Introduction

Systemic sclerosis (SSc) is an autoimmune disorder characterized by vasculopathy and fibrosis of multiple organ systems, with the highest mortality of the rheumatic diseases [1]. The prevalence of depression in patients with SSc is 35 to 65% in cross-sectional studies, as measured by validated depression questionnaires [2–6], and rates of depression are significantly higher than in rheumatoid arthritis [7]. In previous studies using diagnosis codes, the prevalence of depression diagnoses among patients with SSc has been reported as 16% [8] to 22% [7] which is lower than depression prevalence by questionnaire, suggesting that significant depressive symptoms as captured on questionnaires may not be recognized by clinicians, leading to underdiagnosis of depression among patients with SSc.

The purpose of this study was to describe the prevalence of significant depressive symptoms among participants with SSc completing annual depression questionnaires. We aimed to describe rates of clinically diagnosed depression and mental health treatment among individuals with elevated depression scores and to evaluate for associations between demographic and clinical factors in relation to mental health diagnoses and treatment. Additionally, we aimed to evaluate for associations of SSc symptoms and the COVID-19 pandemic with a continuous CESD-R score.

## 2. Methods

### 2.1. Eligibility and Recruitment

The Prospective Registry in Scleroderma at Massachusetts General Hospital (PRISM) is an ongoing longitudinal registry of patients with SSc seen at the Massachusetts General Hospital Rheumatology Clinic. All patients fulfilled the 2013 American College of Rheumatology/European League Against Rheumatism (ACR/EULAR) Classification for SSc. PRISM collects annual clinical assessments and surveys on symptom burden and quality of life measures. For this analysis, we included participants who completed the CESD-R at least once. All participants in PRISM provided written informed consent, data were collected at the time of a routine clinic appointment, and patients completed study questionnaires and clinical measurements at enrollment and annually. The study was approved by the institutional review board. PRISM patients with a baseline visit between March 2015 and April 2022 were included in this analysis, and the sample size was determined by available data at the time of analysis.

### 2.2. Assessments

Participants in PRISM receive questionnaires every 12 months. Depressive symptoms were assessed using CESD-R, which is a structured questionnaire validated to measure depressive symptoms in SSc [9, 10]. Based on validation studies of CESD-R [9, 11], we defined a participant as having possible depression if CESD-R score ≥ 16 and probable depression if CESD-R score ≥ 23.

Patient-reported outcomes measurement information system (PROMIS)-29v2 [12], the health assessment questionnaire disability index (HAQ-DI) [13], and patient global assessment were administered. The modified Rodnan skin score (mRSS) was completed by a physician specializing in SSc at each visit. Echocardiogram and pulmonary function tests were reported if they were included as part of usual clinical care. Symptoms were assessed using the baseline dyspnea index (BDI) [14], the University of California Los Angeles Scleroderma Clinical Trial Consortium Gastrointestinal Tract 2.0 (UCLA SCTC GIT 2.0) [15], and Raynaud's condition score (RCS). During COVID-19 pandemic was defined as all questionnaires completed from February 2020 through May 2022 when data was exported.

### 2.3. Chart Review

Among the subset of participants with CESD-R scores ≥16 for at least one time point, a chart review was conducted by two investigators to abstract mental health diagnoses and treatment over the two years prior to and after the elevated CESD-R score. Standardized terms (“depression,” “anxiety,” “mood disorder,” “psychiatry,” “psychotherapy,” “counseling,” “cognitive behavioral therapy,” “psychotherapeutic drugs,” and “therapist”) were queried in a searchable electronic health record. Reviewers evaluated whether participants were diagnosed with or received treatment for a mood or anxiety disorder within two years before or after the date of the first CESD-R survey. Psychiatric medications were noted regardless of whether the indication was for a mood disorder. Medications were categorized as selective serotonin reuptake inhibitors (SSRI), serotonin-norepinephrine reuptake inhibitors (SNRI), atypical agents, serotonin modulators, tricyclic antidepressants, monoamine oxidase inhibitors, benzodiazepines, and others. Clinician recommendation for counseling, psychotherapy, or psychiatry evaluation was noted.

### 2.4. Statistical Analyses

We reported summary statistics as means with standard deviation, medians with interquartile range (IQR), or number with proportion of the cohort. We assessed the relation of demographic and clinical factors to the prevalence of depression diagnosis or treatment among participants with high depression scores using logistic regression. We examined the association of SSc-related symptoms (BDI, RCS, UCLA SCTC GIT 2.0, and mRSS) and prepandemic versus during the COVID-19 pandemic with CESD-R depression score using quantile regression (CESD-R scores were not normally distributed). In each multivariable-adjusted regression model, we adjusted for the potential confounders based on causal diagrams. Statistical significance was defined as a two-sided *p* value < 0.05. Analyses were performed using SAS software 9.4.

## 3. Results

Of 214 patients enrolled in PRISM as of May 2022, 129 participants completed at least one CESD-R questionnaire and 85 completed a repeat CESD-R questionnaire at 12 months (Table 1). One hundred fifteen participants (89%) identified as female, and the median age was 62 years (IQR: 53-70). One hundred ten participants (85%) identified as White, one (1%) Black, and nine (7%) Asian/Asian-American. Ten participants (8%) reported Hispanic or Latino ethnicity.

Forty-nine (38%) participants were classified as having diffuse SSc, 76 (59%) limited SSc, and four (3%) SSc sine scleroderma (Table 1). The median disease duration was 4.5 years from the first non-Raynaud symptom, and 23% were diagnosed with interstitial lung disease. Among 108 patients who underwent echocardiogram as part of routine clinical care, 17% had concern for pulmonary hypertension with estimated pulmonary artery systolic pressure ≥ 40 mmHg.

In the initial survey, the median CESD-R score was 10 (IQR 5-17); 29% had possible depression (CESD − R ≥ 16), and 16% had probable depression (CESD − R ≥ 23) (Table 2). Median depression scores and proportions of elevated depression scores were similar over three years of annual follow-up (29% CESD − R ≥ 16 at the initial survey (*N* = 129) versus 30% CESD − R ≥ 16 at the 24-month survey (*N* = 43)).

Of 38 participants with possible depression (CESD − R ≥ 16), 23 (61%) were diagnosed with a mood disorder, and 26 (68%) received first-line pharmacotherapy (SSRI, SNRI, atypical antidepressant, serotonin modulator, tricyclic antidepressant, or monoamine oxidase inhibitor). Of 20 participants with probable depression (CESD − R ≥ 23), 13 (65%) were diagnosed with a depressive disorder, and 17 (85%) received first-line pharmacotherapy for major depressive disorder. Two participants were diagnosed with bipolar disorder. Among participants with probable depression (CESD − R ≥ 23), two participants (10%) had no mental health diagnoses and no mental health treatments (pharmacotherapy or psychotherapy). 66% of patients with CESD-R score ≥ 16 had documentation of anxiety in chart review; however, it was challenging to determine whether these symptoms constituted an anxiety disorder or an adjustment to illness and situational factors. Benzodiazepines were prescribed for 26% of participants with CESD − R ≥ 16. Referrals to a psychiatrist, psychologist, or therapist occurred for 47% of participants with possible depression (CESD − R ≥ 16) and 55% of participants with probable depression (CESD − R ≥ 23). Twenty patients (53%) were not referred to mental health specialty care or counseling.

A multivariate logistic regression model was developed to evaluate associations of CESD-R score, age, gender, employment status, race, and ethnicity with mental health diagnosis or treatment among participants with CESD − R ≥ 16. None of the evaluated variables was significantly associated with mental health diagnosis or treatment.

In quantile regression models of SSc symptoms (measured by BDI, RCS, mRSS, and UCLA SCTC GIT 2.0) and CESD-R score, adjusted for age, gender, and disease duration from first non-Raynaud symptoms, dyspnea by BDI and skin involvement by mRSS were associated with higher CESD-R score. Gastrointestinal symptoms measured by UCLA SCTC GIT 2.0 total score and subscales of reflux, distension/bloating, diarrhea, fecal soilage, emotional well-being, and social functioning were also associated with higher CESD-R score (Table 3). Quantile regression models did not demonstrate the association of UCLA SCTC GIT 2.0 constipation subscale, RCS, and pre versus during COVID-19 pandemic with the CESD-R score.

## 4. Discussion

Our finding that 29% of participants had CESD-R scores ≥ 16 is similar to findings from a cross-sectional analysis of 376 Canadian patients with SSc in which 32% had CESD-R scores ≥ 16 [4]. On chart review, we found that 90% of participants with probable depression were clinically diagnosed with and/or treated for a mental health condition, and 85% of these participants received first-line pharmacotherapy for depression, reflecting widespread recognition of mental health problems and provider comfort with managing depression. Interestingly, the rate of benzodiazepine use (26% of patients with elevated depression score) was higher than expected considering significant side effects, safety risks, and risk of dependence with this class of medications [16].

Pharmacotherapy for depression among participants with probable depression (CESD − R ≥ 23) was more common than referral for psychotherapy or mental health specialty care (85% versus 55%, respectively). Multiple randomized clinical trials have demonstrated an additive benefit to psychotherapy with pharmacotherapy as an initial treatment for depression [17]. There are several reasons why referrals and uptake of psychotherapy may be low. Patients with SSc may feel overwhelmed with the frequency of medical appointments and may prioritize other health problems over depression. There are also constraints on the existing mental health infrastructure; in one US study, about two-thirds of primary care physicians reported they could not find outpatient mental health services for patients [18]. We did not find associations between demographic factors and access to mental health care although racial disparities in mental health treatment are well established [19, 20]. We enrolled a low proportion of Black participants (1%) and a low proportion of Hispanic or Latino participants (8%), and rates of mental health treatment were high; thus, our study was likely not powered to detect an association between race and depression treatment.

Dyspnea by BDI, skin involvement by mRSS, and gastrointestinal symptoms by UCLA SCTC GIT 2.0 were associated with higher depression scores. Our findings are consistent with results from a cohort of Iranian patients with SSc, in which dyspnea and gastrointestinal symptoms were associated with higher depression by Beck's depression index [21]. While a relationship between the severity of skin thickening and depression has not been observed in larger studies of clinical correlates of depression [4, 21], we found an association between mRSS scores and CESD-R scores. Body image dissatisfaction related to skin involvement may be a contributing factor; findings from a study of women with systemic sclerosis suggest that depressive symptoms mediate the relationship between body image dissatisfaction and impaired psychosocial function [22]. Additionally, we observed that every UCLA SCTC GIT 2.0 subscale except for constipation was associated with the CESD-R score, consistent with prior reports of association between gastrointestinal and mood symptoms [4, 23, 24]. While unpleasant gastrointestinal symptoms may impair the quality of life and increase the risk for depression, it is possible that perception of gastrointestinal symptoms is exacerbated by depression.

Limitations of our study include a small sample size with a low representation of racial and ethnic minorities. Only English questionnaires were available to participants. There was a high loss to follow-up on subsequent depression surveys; thus, it was not possible to analyze trends in depression over time. In our study, clinicians were alerted to high depression scores, demonstrating the feasibility of a screening questionnaire incorporated into clinical practice. At the same time, this approach likely increased clinician recognition of depression and contributed to higher-than-expected rates of mental health diagnosis and treatment in this cohort. Strengths of our study include a well-characterized longitudinal cohort of patients with SSc with serial clinical assessments and patient-reported outcomes including CESD-R, HAQ-DI, UCLA SCTC GIT 2.0, PGA, RCS, and BDI. The validity of the chart review was enhanced by two independent reviewers.

## 5. Conclusion

In this observational cohort study of SSc patients at a single academic center who completed depression questionnaires, we found that 29% of participants had possible depression, and 16% had probable depression by CESD-R. Among patients with probable depression by questionnaire, we observed high rates of depression diagnoses (90%) and use of first-line pharmacotherapy (85%). Rates of referral for psychotherapy (55%) were lower than rates of pharmacotherapy for depression, highlighting potential areas for improvement in mental health care delivery. Dyspnea, skin involvement, and gastrointestinal symptoms were associated with higher depression scores, suggesting that increased somatic symptom burden contributes to the high prevalence of depression in SSc compared with other rheumatic diseases.

In conclusion, depression is a common comorbidity in patients with SSc, and rheumatologists should consider screening all SSc patients for depression in clinical practice. In line with prior reports, we found associations between dyspnea and gastrointestinal symptoms and depression. Additional research is needed to determine the effects of depression screening on mental health and patient-reported outcomes among patients with SSc.

## Figures and Tables

**Table 1:** Demographic and clinical characteristics of participants completing CESD-R.

Characteristic	All participants (*N* = 129)	Participants with CESD − R ≥ 16 at first survey (*N* = 38)
	*N* (%) or median (IQR)	
Gender		
Female	115 (89)	35 (92)
Age (years)	62 (53-70)	59 (49-68)
Race		
Black	1 (1)	0
Asian-American	9 (7)	1 (3)
White	110 (85)	35 (92)
Others, more than one race, or unknown	9 (7)	3 (8)
Ethnicity		
Hispanic or Latino	10 (8)	3 (8)
Non-Hispanic or Latino	118 (92)	35 (92)
Employment status		
Working, student, or homemaker	50 (39)	16 (42)
Disabled or retired	40 (31)	8 (21)
Unknown	39 (30)	14 (37)
Disease duration from first Raynaud's symptom (years) (*N* = 125)	9 (4-31)	9 (2-18)
Disease duration from first non-Raynaud's symptom (years) (*N* = 125)	5 (2-11)	4 (2-11)
Systemic sclerosis subtype		
Diffuse cutaneous	49 (38)	16 (42)
Limited cutaneous	76 (59)	21 (55)
Sine scleroderma	4 (3)	1 (3)
Autoantibodies (if ever positive):		
Anticentromere (*N* = 32)	15 (12%)	3 (8%)
Anti-Scl70 (*N* = 97)	23 (18%)	3 (8%)
RNA polymerase III (*N* = 112)	4 (3%)	2 (5%)
Interstitial lung disease^∗^ (*N* = 127)	30 (23)	15 (40)
Functional vital capacity (FVC) percent predicted (*N* = 109)	94 (78-105)	89 (69-101)
Diffusing capacity of the lungs for carbon monoxide (DLCO) percent predicted (*N* = 107)	72 (50-86)	64 (50-79)
Pulmonary hypertension estimated by echocardiogram^∗∗^ (*N* = 108)	18 (17)	6 (16)
Pulmonary hypertension confirmed by right heart catheterization (*N* = 10)	8 (6%)	2 (5%)
Baseline dyspnea index (*N* = 73)	3 (1-4)	4 (2-8)
History of digital ischemic ulcers	11 (9%)	3 (8%)
Modified Rodnan skin score (*N* = 123)	4 (2-10)	6 (2-17)
Gastrointestinal manifestations‡	113 (88%)	36 (95%)
UCLA SCTC GIT 2.0 total score (*N* = 128)	0.5 (0.1-0.8)	0.8 (0.4-1.1)
History of scleroderma renal crisis (*N* = 125)	1 (1%)	0
Immunomodulatory medication use at enrollment		
Mycophenolate or mycophenolic acid	48 (37%)	19 (50%)
Prednisone	19 (15%)	4 (10%)
Methotrexate	13 (10%)	4 (10%)
Hydroxychloroquine	11 (9%)	3 (8%)
Other	3 (2%)	2 (2%)
Patient global assessment	3 (2-6)	6 (4-7)
Health assessment questionnaire disability index (*N* = 127)	0.6 (0.1-1.4)	1.4 (0.8-1.9)
PROMIS-29 Profile v2.0 (*N* = 116)		
Physical function	43 (37-57)	39 (32-42)
Anxiety	51 (40-60)	61 (56-65)
Depression	49 (41-56)	57 (54-62)
Fatigue	55 (46-63)	63 (59-67)
Sleep	51 (46-56)	58 (52-64)
Activities	51 (44-64)	44 (37-44)
Pain	54 (42-61)	61 (57-65)

^∗^By physician assessment at study intake, not all patients had high-resolution chest CT available in PRISM. ^∗∗^Transthoracic echocardiogram with estimated pulmonary artery systolic pressure ≥ 40. ‡Patients with any of the following gastrointestinal symptoms on baseline physician assessment: gastroesophageal reflux disease, esophageal dysmotility, esophageal stricture, small bowel hypomotility, or small intestine bacterial overgrowth. Percentages calculated with 129 as denominator, irrespective of missing data.

**Table 2:** Depression scores on annual CESD-R questionnaires.

CESD-R score	Baseline (*N* = 129)	12 months (*N* = 82)	24 months (*N* = 43)	36 months (*N* = 12)
Median [IQR]	10 [5-17]	10.5 [3-16]	12 [4-17]	13 [10-17]
Mean (SD)	12.2 (10.1)	11.3 (9.7)	12.0 (9.4)	14.8 (8.5)
CESD − R ≥ 16	38 (29%)	23 (28%)	13 (30%)	3 (25%)
CESD − R ≥ 23	20 (16%)	11 (13%)	4 (9%)	2 (17%)

**Table 3:** Associations of SSc symptom burden and COVID-19 pandemic with CESD-R score.

Characteristic	CESD-R score	
	Beta coefficient (confidence interval)	*P*
Baseline dyspnea index^∗^	2.7 (1.5-3.8)	<0.01
UCLA SCTC GIT 2.0 total score	9.5 (4.8-14.1)	<0.01
UCLA SCTC GIT 2.0 reflux subscale	7.1 (2.5-11.8)	<0.01
UCLA SCTC GIT 2.0 distension/bloating^∗∗^	4.1 (1.4-6.8)	<0.01
UCLA SCTC GIT 2.0 diarrhea^‡^	6.3 (2.8-9.9)	<0.01
UCLA SCTC GIT 2.0 fecal soilage^∗∗^	5.0 (0.1-9.9)	0.05
UCLA SCTC GIT 2.0 constipation	2.6 (-0.7-5.9)	0.11
UCLA SCTC GIT 2.0 emotional well-being^∗∗^	6.9 (2.7-11.1)	<0.01
UCLA SCTC GIT 2.0 social function^‡^	10.2 (5.1-15.3)	<0.01
Raynaud's condition score	0.8 (-0.1-1.7)	0.09
Modified Rodnan skin score	0.4 (0.1-0.6)	<0.01
Timepoint pre versus during COVID-19 pandemic	0.8 (-4.7-6.3)	0.76

Quantile regression models comparing median values, adjusted for age, gender, and disease duration since first non-Raynaud's symptoms. 128 participants included in each model unless otherwise indicated. ^∗^Missing dyspnea index data from 62 participants, *N* = 67. ^∗∗^Missing data from 1 participant, *N* = 127. ‡Missing data from 2 participants, *N* = 126.

## Data Availability

Deidentified data may be requested from study authors.

## Supplementary Materials

Supplementary Materials STROBE guidelines for cross-sectional studies are attached with notes indicating where the requirements are found in the manuscript.
