# Supplementary material for: Clinically Recognized Depression and Mental Health Treatment in a Single Center Cohort of Patients with Systemic Sclerosis
Source: Int J Rheumatol. 2023 Dec 19;2023:6141790. doi: 10.1155/2023/6141790 (PMC10751161; doi:10.1155/2023/6141790)
Supplement: Supplementary Materials — STROBE guidelines for cross-sectional studies are attached with notes indicating where the requirements are found in the manuscript. [file 6141790.f1.doc]

STROBE Statement—Checklist of items that should be included in reports of ***cross-sectional studies***

|  | Item No | Recommendation |
| --- | --- | --- |
| **Title and abstract** | 1 | (*a*) Indicate the study’s design with a commonly used term in the title or the abstract  *- described cross-sectional study as part of a longitudinal cohort in conclusion of abstract (line 43)* |
|  |  | (*b*) Provide in the abstract an informative and balanced summary of what was done and what was found  *- described methods and described positive and negative findings* |
| Introduction | | |
| Background/rationale | 2 | Explain the scientific background and rationale for the investigation being reported  *-lines 48-56 of introduction describe prior data and rationale for study design* |
| Objectives | 3 | State specific objectives, including any prespecified hypotheses *-lines 58-64* |
| Methods | | |
| Study design | 4 | Present key elements of study design early in the paper *-larger cohort described lines 68-72.* |
| Setting | 5 | Describe the setting, locations, and relevant dates, including periods of recruitment, exposure, follow-up, and data collection *-line 68-69, 76-77* |
| Participants | 6 | Give the eligibility criteria, and the sources and methods of selection of participants  *-lines 70-73* |
| Variables | 7 | Clearly define all outcomes, exposures, predictors, potential confounders, and effect modifiers. Give diagnostic criteria, if applicable *-lines 80-117* |
| Data sources/ measurement | 8* | For each variable of interest, give sources of data and details of methods of assessment (measurement). Describe comparability of assessment methods if there is more than one group *-lines 80-109* |
| Bias | 9 | Describe any efforts to address potential sources of bias *-two investigators reviewed charts, lines 97-99* |
| Study size | 10 | Explain how the study size was arrived at *-convenience sample size, described lines 76-78* |
| Quantitative variables | 11 | Explain how quantitative variables were handled in the analyses. If applicable, describe which groupings were chosen and why  *-lines 113-121* |
| Statistical methods | 12 | Describe all statistical methods, including those used to control for confounding  *-lines 112-121* |
|  |  | Describe any methods used to examine subgroups and interactions *-none* |
|  |  | Explain how missing data were addressed *-footnote of table 1, line 312* |
|  |  | If applicable, describe analytical methods taking account of sampling strategy *-NA* |
|  |  | Describe any sensitivity analyses *-NA* |
| Results | | |
| Participants | 13* | Report numbers of individuals at each stage of study—eg numbers potentially eligible, examined for eligibility, confirmed eligible, included in the study, completing follow-up, and analysed *-inclusion in this depression study vs larger pool of PRISM participants described in lines 124-125* |
|  |  | Give reasons for non-participation at each stage *-all due to not having filled out depression questionnaire* |
|  |  | Consider use of a flow diagram |
| Descriptive data | 14* | Give characteristics of study participants (eg demographic, clinical, social) and information on exposures and potential confounders *-table 1, line 311* |
|  |  | Indicate number of participants with missing data for each variable of interest *-table 1, line 311* |
| Outcome data | 15* | Report numbers of outcome events or summary measures *-lines 137-157* |
| Main results | 16 | (*a*) Give unadjusted estimates and, if applicable, confounder-adjusted estimates and their precision (eg, 95% confidence interval). Make clear which confounders were adjusted for and why they were included *-Table 3 page 16* |
|  |  | (*b*) Report category boundaries when continuous variables were categorized *-Table 1 page 13* |
|  |  | (*c*) If relevant, consider translating estimates of relative risk into absolute risk for a meaningful time period *-NA* |
| Other analyses | 17 | Report other analyses done—eg analyses of subgroups and interactions, and sensitivity analyses *-NA* |
| Discussion | | |
| Key results | 18 | Summarise key results with reference to study objectives *-lines 174-182* |
| Limitations | 19 | Discuss limitations of the study, taking into account sources of potential bias or imprecision. Discuss both direction and magnitude of any potential bias *-lines 211-221* |
| Interpretation | 20 | Give a cautious overall interpretation of results considering objectives, limitations, multiplicity of analyses, results from similar studies, and other relevant evidence |
| Generalisability | 21 | Discuss the generalisability (external validity) of the study results *-lines 217-220* |
| Other information | | |
| Funding | 22 | Give the source of funding and the role of the funders for the present study and, if applicable, for the original study on which the present article is based *-lines 13-15* |

*Give information separately for exposed and unexposed groups.

**Note:** An Explanation and Elaboration article discusses each checklist item and gives methodological background and published examples of transparent reporting. The STROBE checklist is best used in conjunction with this article (freely available on the Web sites of PLoS Medicine at http://www.plosmedicine.org/, Annals of Internal Medicine at http://www.annals.org/, and Epidemiology at http://www.epidem.com/). Information on the STROBE Initiative is available at www.strobe-statement.org.
